# The MC4R agonist, setmelanotide, is associated with an improvement in hypercapnic chemosensitivity and weight loss in male mice

**DOI:** 10.1016/j.resp.2024.104370

**Published:** 2024-11-13

**Authors:** Athena Rivera, Sarah N. Framnes-DeBoer, Deanna M. Arble

**Affiliations:** Department of Biological Sciences, Marquette University, WI, USA

**Keywords:** Pair-feeding, High-fat diet, Hypercapnic ventilatory response, Obesity, Breathing

## Abstract

Obesity increases the risk of respiratory diseases that reduce respiratory chemosensitivity, such as Obesity Hypoventilation Syndrome and sleep apnea. Recent evidence suggests that obesity-related changes in the brain, including alterations in melanocortin signaling via the melanocortin-4 receptor (MC4R), may underly altered chemosensitivity. Setmelanotide, an MC4R agonist, causes weight loss in both humans and animal models. However, it is unknown the extent to which setmelanotide affects respiratory chemosensitivity independent of body weight loss. The present study uses diet-induced obese, male C57bl/6 J mice to determine the extent to which acute setmelanotide treatment affects the hypercapnic ventilatory response (HCVR). We find that ten days of daily setmelanotide treatment at 1 mg/kg, but not 0.2 mg/kg, is sufficient to cause weight loss and increase HCVR. In a separate group of animals, we find that we can emulate setmelanotide’s effect on weight loss by restricting daily calories to match the hypophagia triggered by setmelanotide. These pair-fed animals exhibit improvements in HCVR similar to those who receive setmelanotide. We conclude that acute treatment with setmelanotide is as effective as weight loss at improving respiratory hypercapnic chemosensitivity.

## 1. Introduction

Respiratory diseases such as obesity hypoventilation syndrome (OHS) and sleep apnea are highly associated with obesity and significantly affect one’s quality of life and mortality (Young et al., 2008; Lopez et al., 2008; Moyer et al., 2001). Individuals with OHS are principally diagnosed through high body mass index (BMI), high levels of arterial carbon dioxide, and a sleep study to quantify carbon dioxide production (Vidya and Genta, 2021). OHS is also associated with shallow breathing and a reduction in the ability to maintain blood gas homeostasis via respiratory chemosensitivity, which reflects a problem in the central drive to breathe (Vidya and Genta, 2021; Olson and Zwillich, 2005). The most prevalent form of sleep apnea is Obstructive Sleep Apnea (OSA). Like OHS, OSA is significantly associated with obesity (Lopez et al., 2008; Peppard, 2000). There is growing evidence that many individuals with OSA may have undiagnosed central apneic events, as evidenced by a reduction in chemosensitivity observed in 40–70% of individuals with OSA (Young et al., 2008; Lopez et al., 2008).

Evidence from rodent models suggests that central changes in the brain, consistent with obesity, may underly altered chemosensitivity (Flak et al., 2017; Polotsky et al., 2001; Polak et al., 2013; Ribeiro et al., 2013). For example, melanocortin-4 receptors (MC4R), involved in body weight regulation (Farooqi et al., 2003; Bassi et al., 2015; Doulla et al., 2014), modulate chemosensitivity. Global knock out of MC4R in a mouse results in obesity and reduced chemosensitivity, implying the potential neuromediation of breathing through MC4R (Bassi et al., 2015). Genetic ablation of the MC4R ligand precursor, proopiomelanocortin (POMC), similarly affects the chemosensitivity of mice (Bassi et al., 2015). Moreover, patients with MC4R deficiency exhibit obesity and respiratory diseases, further insinuating a potential role of MC4R in obesity-related respiratory disorders (Doulla et al., 2014). It remains unknown if MC4R is a viable target for improving the obesity-associated chemosensitivity observed in humans.

The FDA has approved a drug which acts on MC4R for weight loss in individuals with POMC or leptin deficiency (Collet et al., 2017; Pressley et al., 2022; Clément et al., 2018). This drug, called setmelanotide, stimulates an increase in α-MSH that acts on MC4R, potentially at the paraventricular nucleus and lateral hypothalamus which are known for their overlapping roles in body weight regulation and breathing modulation (Thaler et al., 2012; McNay et al., 2012; Markham, 2021; Kievit et al., 2013; Chen et al., 2015; Ryan, 2020; Haws et al., 2020). While setmelanotide causes a ~10% decrease in body weight (Farooqi et al., 2003; Collet et al., 2017; Pressley et al., 2022; Haqq et al., 2022), the extent to which setmelanotide affects chemosensitivity remains unknown. This remains unknown, in part, because weight loss alone improves chemosensitivity (Bhammar et al., 2016; Littleton, 2012; Piper and Grunstein, 2010; Ray et al., 1983; Marain et al., 2022; Aaron et al., 2004). Controlled studies are needed to determine the extent to which setmelanotide affects chemosensitivity above and beyond its effect on weight loss.

In the present study, we sought to determine the extent to which setmelanotide, an MC4R agonist, affects chemosensitivity independent of weight loss. To test this, we used diet-induced obese (DIO) male mice that exhibit a reduction in hypercapnic chemosensitivity. Mice were randomly assigned to setmelanotide or vehicle treatment and allowed *ad libitum* access to food. To control for weight loss, an additional group underwent caloric restrtiction (*i.e.,* pair-feeding) to match the hypophagia and eventual weight loss caused by setmelanotide. We find that acute treatment with setmelanotide improves hypercapnic chemosensitivity of obese, male mice in a dose-dependent manner. The ability of setmelantotide to improve chemosensitivity occurs secondary to weight loss, as untreated animals with similar weight loss exhibited similar improvments in hypercapnic chemosensitivity.

## 2. Methods

### 2.1. Animals

16-week-old, male C57BL/6 J wildtype (WT) and diet-induced obese (DIO) WT mice were ordered from Jackson Laboratory (Bar Harbor, ME). WT lean mice were maintained on a standard chow diet (21.55% kcal from fat, PicoLab 5058). DIO mice were maintained on a hypercaloric diet (60% kcal from fat, Teklad, TD.06414) for at least 12 weeks before experimentation. Mice were housed in 12 L:12D light conditions with lights on at 8 am and off at 8 pm. Zeitgeber Time (ZT) 0 corresponds to lights on and ZT 12 corresponds to lights off. All studies were reviewed, approved by, and performed according to the guidelines of the Institutional Animal Care and Use Committee at Marquette University (Milwaukee, WI).

### 2.2. Vehicle and setmelanotide injections

All animals were randomized into study groups and received intraperitoneal injections (either vehicle or setmelanotide) 1 – 1.5 hour before dark onset (ZT 10.5 – 11.5). Setmelanotide (Millipore Sigma TA9H98DC9B7C) was dissolved in a solution of 0.9% saline, 2% heat-inactivated normal mouse serum, and 0.5% DMSO. Setmelanotide was given daily for up to 14 days at a dose of 0.2 mg/kg or 1.0 mg/kg. Vehicle injections comprised of 0.9% saline, 2% heat-inactivated normal mouse serum, and 0.5% DMSO and administered daily for up to 14 days.

### 2.3. Pair-feeding/caloric restriction

To emulate the hypophagia of setmelanotide, animals in the vehicle pair-fed group had their calories restricted to match the daily calories consumed by the setmelanotide group. All animals and food were weighed daily at ZT 6–7. At that time, pair-fed animals received measured, restricted food. If leftover food was found in the cages of the pair-fed animals when new food was to be distributed, that leftover food was removed before to the new food was added.

### 2.4. Whole-body plethysmography

Baseline, eucapnic ventilation and the hypercapnic ventilatory response (HCVR) was measured at ZT 0–2 in freely moving, awake mice using whole-body plethysmography (SCIREQ / EMKA Technologies) as previously described (Jones et al., 2023). Briefly, animals were acclimated to the plethysmograph chamber for 30 min. Eucapnic (21% O_2_ and 79% N_2_) breathing measurements were taken during a 4 min period while the animal was visually confirmed in the awake state. Afterwards, 5 min bouts of CO_2_ challenges at 5% (5% CO_2_, 21% O_2_ and 74% N_2_), 8% (8% CO_2_, 21% O_2_ and 71% N_2_), and 3% (3% CO_2_, 21% O_2_ and 76% N_2_) were provided respectively with a 5 min period of eucapnic exposure in between each challenge. At all times, animals were confirmed in the awake state by visual inspection. Ventilation measures included inspiration, expiration, minute volume, tidal volume, enhanced pause, peak inspiratory flow, peak inspiratory flow, breathing frequency, and expiration volume to inspiration volume ration. The ventilatory response to hypercapnia was recorded and was used to calculate hypercapnic ventilatory response (slope of minute volume per CO_2_).

### 2.5. Body weight and glucose tolerance measurements

All animals had food weighed daily at ZT 6–7. Food intake was calculated by weighing the food daily and subtracting the current weight of the food from the weight of the previous day. The glucose tolerance test (GTT) was performed on the 10th day of the study. At ZT 0, mice were transferred to clean cages and fasted for 4 hours. Blood glucose levels were assessed from the tail via a commercially available glucometer at 0, 15, 30, 60, and 120 minutes following an interparietal injection 2.0 mg/kg glucose.

### 2.6. Statistical analysis

All statistics were performed using Graph Pad Prism 10. All values are reported as means ± SEM and were analyzed by 1-way ANOVA, 2- way ANOVA, or t-test, along with either Tukey’s or Sidak’s multiple comparisons test, when appropriate.

## 3. Results

### 3.1. Setmelanotide (1 mg/kg dose) improves the hypercapnic chemoreflex of obese, male mice

**Fig. 1.**
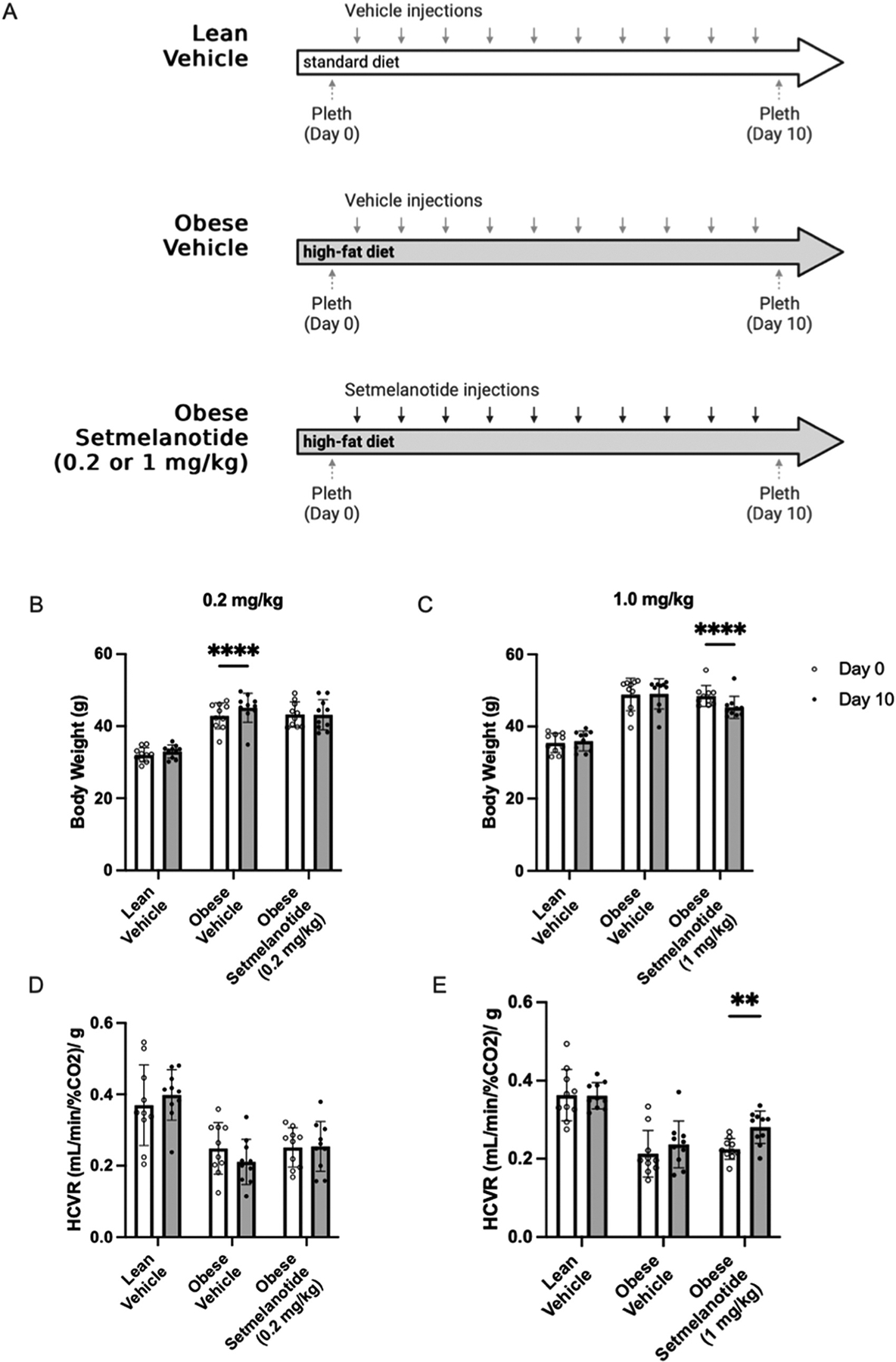
Setmelanotide (1 mg/kg) improves the hypercapnic ventilatory response and causes weight loss. **A**) Schematic of protocols used. Top panel indicates groups used with all interventions. **B**) After 10 days, 0.2 mg/kg of setmelanotide prevented weight gain in DIO mice. Repeated Measures, two-way ANOVA with Sidak’s multiple comparison test, **** p < 0.0001. **C**) By day 10, 1.0 mg/kg of setmelanotide caused weight loss in DIO mice. Repeated measures, two-way ANOVA with Sidak’s multiple comparison test, **** p < 0.0001. **D**) 0.2 mg/kg of setmelanotide did not affect the hypercapnic ventilatory response (HCVR) of obese mice. **E**) 1.0 mg/kg of setmelanotide increased HCVR from Day 0 to Day 10. Repeated Measures Two-way ANOVA with Sidak’s multiple comparison test, ** p < 0.01.

Diet-induced obese (DIO) male mice were given daily IP injections of setmelanotide at 0.2 mg/kg or 1.0 mg/kg doses (Fig. 1 A) to produce differential effects on body weight (Bischof et al., 2016) (Supplemental Figure 1). As expected, setmelanotide prevented weight gain or led to weight loss depending on the treatment dose (Collet et al., 2017; Haqq et al., 2022). Acute treatment with setmelanotide at 0.2 mg/kg prevented weight gain in DIO male mice (Fig. 1 B and C), whereas 1.0 mg/kg of setmelanotide caused 3.7 ± 1.6 g of weight loss (Fig. 1 C). Neither dose of setmelanotide affected glucose tolerance when compared to DIO mice receiving vehicle (Supplemental Figure 2). After setmelanotide treatment, the hypercapnic ventilatory response (HCVR) was assessed using whole-body plethysmography. Whereas the low-dose of setmelanotide (0.2 mg/kg) had no effect on HCVR (Fig. 1 D), the high-dose of setmelanotide (1 mg/kg) significantly improved the chemosensitivity of DIO mice at Day 10 compared to their Day 0 HCVR (Fig. 1 E).

During the low-dose treatment, eucapnic minute ventilation increased over the course of the study in all DIO mice (Supplemental Figure 3 A, significant effect of time, p < 0.001). However, eucapnic minute ventilation decreased over the course of the study in all DIO mice in the high-dose study (Supplemental Figure 3 D, significant effect of time, p < 0.001). These changes in minute ventilation were predominantly due to an increase in tidal volume during the low-dose study (Supplemental Figure 3 C, significant effect of time, p < 0.0001) and a decrease in frequency during the high-dose study (Supplemental Figure 3 E, significant effect of time, p < 0.0001). There were no significant interaction effects between study duration and treatment group at either dose, suggesting that the observed increases in HCVR within the setmelanotide group were not due to changes in eucapnic breathing.

Overall, ten days of daily setmelanotide treatment at 1 mg/kg dose is sufficient to cause weight loss and an improvement in HCVR.

### 3.2. Setmelanotide-induced weight loss can be mirrored by reducing the caloric intake of DIO mice

**Fig. 2.**
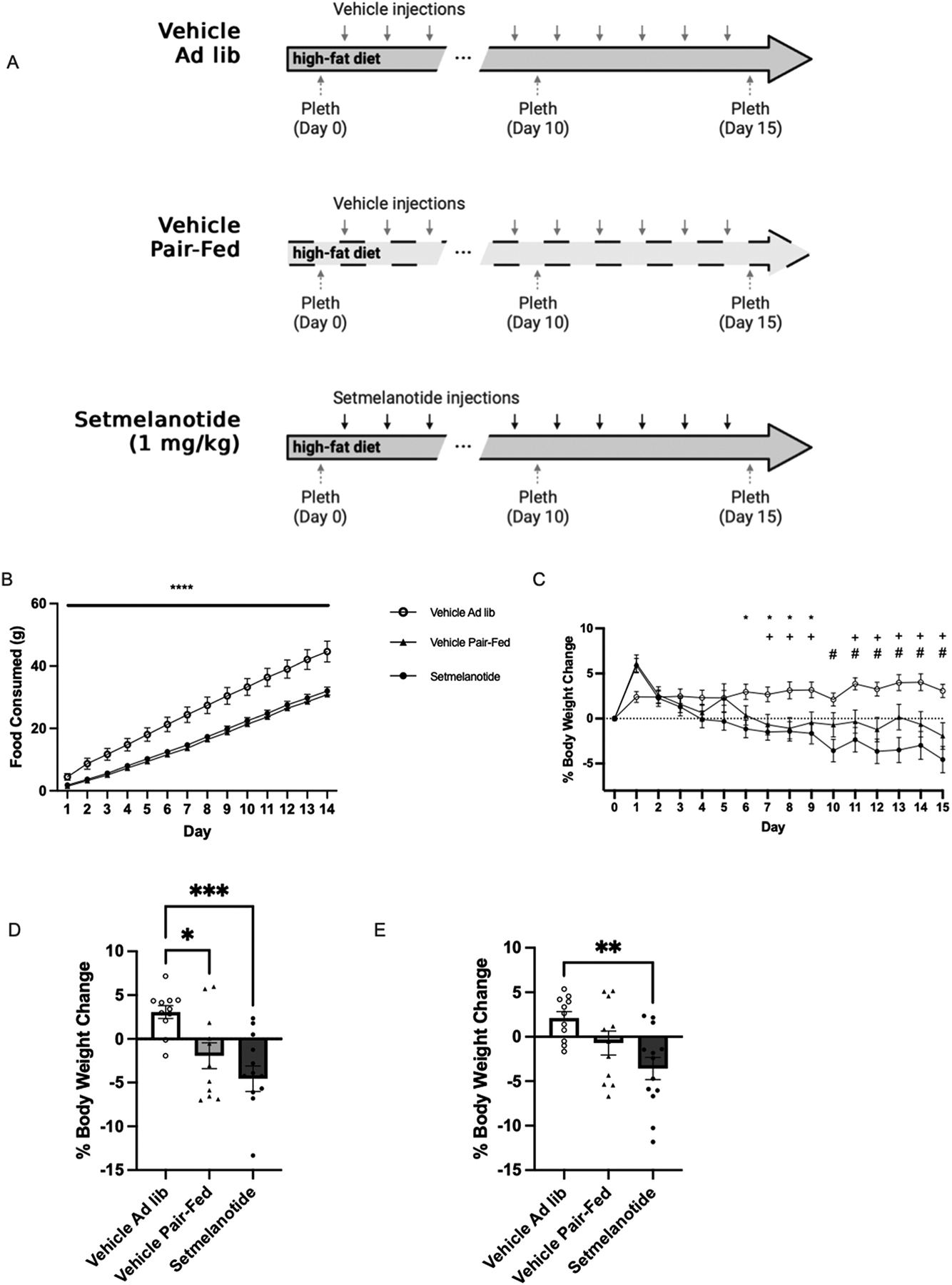
Matching the hypophagia of setmelanotide (1 mg/kg) treatment for 15 days results in similar weight loss. A) Schematic of protocols used. Top black line is the setmelanotide (1.0 mg/kg) ad libitum group, the middle grey line is the vehicle ad libitum group, and the last grey dashed line is the vehicle pair-fed animals. B) Total food consumed significantly differed between the Pair-Fed and Setmelanotide group compared to the Vehicle *ad libitum group*. Two-way ANOVA with Tukey’s multiple comparison test, **** p < 0.0001. C) Percent changes in body weight. Two-way ANOVA with Tukey’s multiple comparison test, for Vehicle ad libitum vs Setmelanotide, * p < 0.01, # p < 0.001; for Vehicle ad libitum vs Vehicle Pair-fed, + p < 0.01. D) Percent body weight change at Day 10. One-way ANOVA with Tukey’s multiple comparison test, * p < 0.05, and *** p < 0.001 E) Percent body weight change Day 15. One-way ANOVA with Tukey’s multiple comparison test, ** p < 0.01.

Setmelanotide leads to weight loss by causing a reduction in caloric consumption and an increase in energy expenditure (Ryan, 2020; Baldini and Phelan, 2019). To determine if the weight loss from setmelanotide can be induced by caloric restriction alone, male DIO mice were randomized into three groups: (1) a Vehicle group, consisting of mice maintained on an *ad libitum* high-fat diet (2) a Setmelanotide group, consisting of mice maintained on an *ad libitum* high-fat diet that received daily injections of setmelanotide (1.0 mg/kg), and (3) a Pair-Fed group, consisting of mice calorically-restricted to match the hypophagia triggered by setmelanotide (Fig. 2 A). All mice in the Vehicle and Pair-Fed groups received daily vehicle injections.

As expected, animals in the Setmelanotide group ate significantly less than the Vehicle group (Fig. 2 B). The hypophagia of the Setmelanotide group dictated the caloric restriction of the Pair-Fed group. As a result, animals in the Pair-Fed group experienced a reduction in body weight comparable to setmelanotide (Fig. 2 C). The Setmelanotide and Pair-Fed groups, experienced significant changes in body weight by Day 7 that was maintained for the duration of the study (Fig. 2 C). Setmelanotide was significantly different at day 6 compared to the ad libitum vehicle (Supplemental Figure 4). At the time of plethysmograph testing (Day 10 and 15), animals in the Setmelanotide and Pair-Fed groups lost a similar percentage of their initial body weight (Fig. 2 D and E). While weight loss in the Setmelanotide group was statistically different from the Vehicle group at both testing days, the Pair-Fed group was only statistically different from the Vehicle group at Day 10, possibly due to higher weight loss variability in the Pair-Fed group. Overall, acute caloric restriction of a high-fat diet is sufficient to match weight loss induced by setmelanotide.

### 3.3. Setmelanotide (1 mg/kg) treatment improves the hypercapnic chemoreflex similar to weight loss alone

**Fig. 3.**
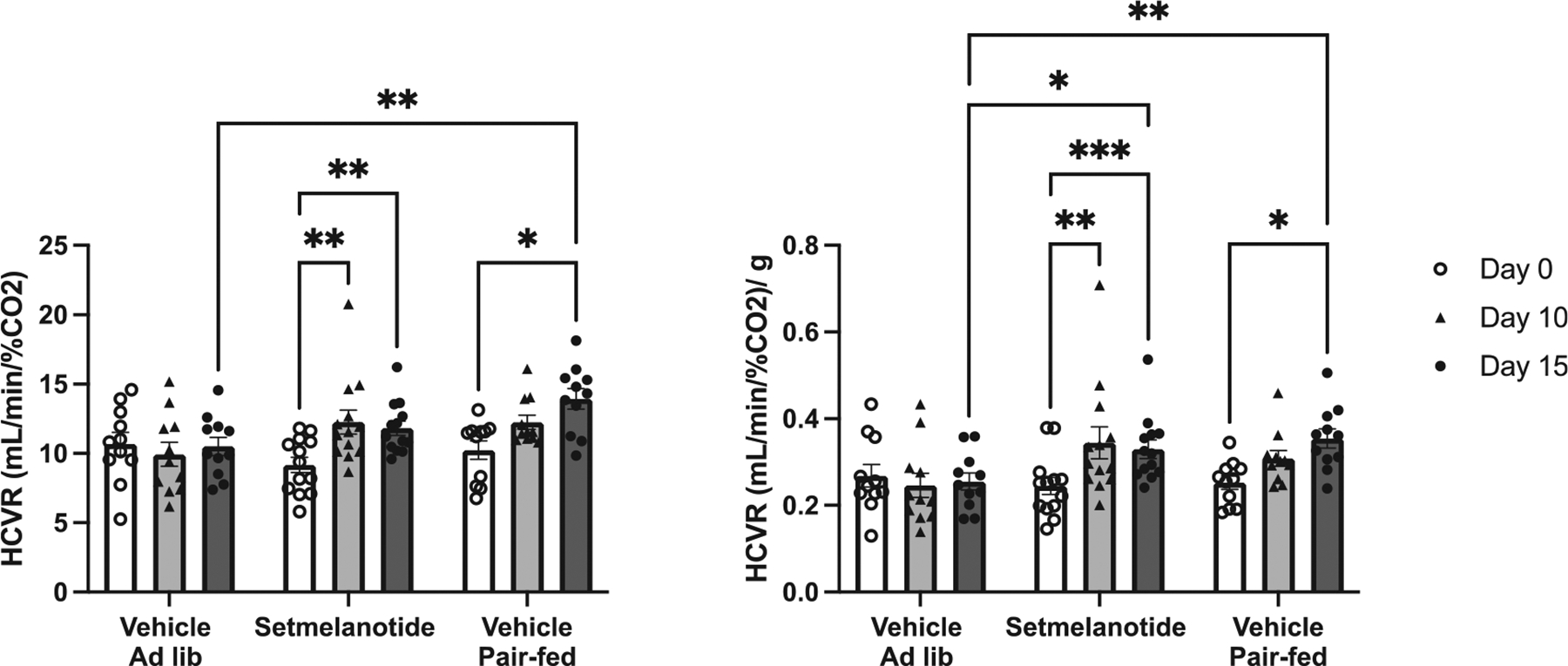
HCVR increased in animals that received setmelanotide, similar to that of the Pair-Fed group. **A**) HCVR from Day 0 to Day 15 was calculated for each group and is represented as the slope of minute ventilation per percent CO_2_ exposure (*e.g.,* ml/min/ %CO_2_) HCVR. Two-way ANOVA with Tukey’s multiple comparison test, * p < 0.05, ** p < 0.01, and *** p < 0.001 **B**) Normalized HCVR. Two-way ANOVA with Tukey’s multiple comparison test, * p < 0.05, ** p < 0.01 and *** p < 0.001.

Considering that weight loss is known to improve chemosensitivity (Anandam et al., 2013), we next determined the extent to which setmelanotide (1.0 mg/kg) affects HCVR beyond weight loss alone. As expected, the hypercapnic chemosensitivity of mice in the Vehicle group was similar to the reduction we observed in our initial study (Fig. 1) and did not change over the duration of the study (Fig. 3). In agreement with our dosing study, we found that setmelanotide (1.0 mg/kg) increased animals’ HCVR at day 10 and 15 (Fig. 3 B) during which time significant weight loss occurred (Fig. 2 A). Mice experiencing weight loss in the Pair-Fed group also exhibited improvements in HCVR at Day 10 and 15 (Fig. 3). To determine how HCVR changes with weight, a normalized HCVR is calculated. At Day 15, both Setmelanotide and Pair-Fed groups experienced similar weight loss, statistically different from the Vehicle group (Fig. 2 A). The normalized HCVR of the Setmelanotide and Pair-Fed groups followed a similar pattern where both groups had a higher normalized HCVR compared to their Day 0 measurements but were similar to each other (Fig. 3 A and B). By Day 15, the normalized HCVR of both the Setmelanotide and Pair-Fed groups were greater than the Vehicle group (Fig. 3 B). Eucapnic minute ventilation and tidal volume did not exhibit any differences (Supplemental Figure 5 A and B). However, eucapnic frequency decreased over the course of the experiment in both the Pair-Fed and Setmelanotide groups (Supplemental Figure 5 C, significant effect of time, p < 0.0001).

## 4. Discussion

Obesity is a major contributing factor to respiratory disease. The reasons for this are multifactorial. In the case of OSA and central sleep apnea, obesity affects lung mechanics, endocrinology, as well as the central control of breathing (Framnes and Arble, 2018). While weight loss is effective at improving respiratory physiology, maintaining long-term weight loss is often difficult. This drives the need to find alternative treatments for obesity-related respiratory diseases that target the underlying cause of the pathophysiology.

Several studies have postulated that obesity-related changes in the brain may contribute to respiratory pathophysiology including a reduction in chemosensitivity. These obesity-related changes may include alterations in the leptin hormone which can stimulate POMC-expressing neurons and subsequently affect MC4R stimulation. Indeed, leptin signaling is an important modulator of ventilatory drive (Framnes and Arble, 2018; Arble et al., 2019; Polotsky et al., 2012; Do et al., 2020) and genetic knockout of POMC or MC4R leads to a reduction in hypercapnic chemosensitivity (Bassi et al., 2015; Doulla et al., 2014). However, it is challenging to isolate the extent to which neurological manipulations alone affect breathing as many of these manipulations also affect body weight. Weight loss itself, achieved through a reduction in calories for example, can also effectively improve HCVR (Olson and Zwillich, 2005; Rochester and Enson, 1974). Therefore, it remains unclear whether targeting obesity-related circuitry provides any additional benefit to respiratory physiology beyond those achieved through weight loss alone.

Here we determine the extent to which setmelanotide, a MC4R agonist, improves hypercapnic chemosensitivity beyond its effect on weight loss. We do this by treating diet-induced obese (DIO) C57bl6/J (B6) male mice with daily setmelanotide and comparing their hypercapnic chemosensitivity to that of an un-treated DIO group that is calorically restricted to match the weight loss observed in the animals receiving setmelanotide.

In agreement with previous studies, we find that 1.0 mg/kg of setmelanotide is sufficient to cause weight loss (Wabitsch et al., 2022; Wang et al., 2023) and it does so partly through a reduction in caloric intake (Kievit et al., 2013; Chen et al., 2015). While several rodent and clinical studies indicate that setmelanotide increases energy expenditure (Chen et al., 2015; Bischof et al., 2016; Clemmensen et al., 2015), we were unable to assess energy expenditure in the present study. However, we find that we can largely mirror the short-term (*e.g.*, < 2 weeks) effects of setmelanotide on weight loss through caloric restriction. This suggests that, at least initially, setmelanotide primarily effects body weight regulation through changes in food intake.

Like prior work in obese animals (Polotsky et al., 2001; O’Donnell et al., 1999; Wei et al., 2020), our data demonstrate that obesity is associated with a decrease in HCVR. Notably, many animal studies normalize HCVR to the animal’s body mass. This enables one to determine if ventilatory drive is rising to meet the expected demands of body mass. To our knowledge, this is the first study to demonstrate that setmelanotide improves the hypercapnic chemosensitivity. The present results indicate that setmelanotide increases chemosensitivity of DIO mice secondary to weight loss. Low dose (0.2 mg/kg) prevented weight gain in DIO mice, but did not cause weight loss. This low dose also did not affect the HCVR. The high dose (1 mg/kg) did cause weight loss and was associated with an increase in HCVR. However, the same weight loss achieved through caloric restricted led to similar improvements in HCVR. We interpret these results to mean that setmelanotide by itself has no added benefit to the HCVR of DIO male mice beyond its effect on weight loss.

How setmelanotide leads to weight loss is an ongoing research question. Setmelanotide has been proposed to lead to weight loss via an increase in energy expenditure and a reduction in food intake via MC4R stimulation of neurons within the paraventricular nucleus (PVN) and lateral hypothalamus (LH). Both the PVN and LH have been implicated in the modulation of breathing (Horiuchi et al., 2009; Williams and Burdakov, 2008; Fukushi et al., 2019) and both are pivotal to body weight regulation (López-Ferreras et al., 2018; Maejima et al., 2019; Schwartz et al., 2000).

However, our data is not without limitations and caution should be used when translating the present findings to the clinic. Since the goal of the present study was to determine how setmelanotide affects chemosensitivity in the context of obesity, we specifically selected an animal model with diet-induced obesity to better mirror the clinical population. While the male C57BL/6 J mouse is a well-used model for translational obesity research (Yang et al., 2014), female C57BL/6 J mice are largely resistant to DIO (Yang et al., 2014; Tortoriello et al., 2007). We elected not to include female mice in the present study for this reason, and this is a notable limitation when considering how this research applies to the clinic. Indeed, while using male mice enables DIO experimental manipulation, this poses a significant limitation due to the known sex differences in ventilatory responses (Wenninger et al., 2009; Tallon et al., 2020). That being said, current clinical data does not suggest that sex affects the ability of setmelanotide to cause weight loss (Setmelanotide (Imcivree), 2023).

Central-acting drugs that are intended for breathing regulation have emerged as potential respiratory disease treatments. These drugs primarily target neural circuits that modulate respiration and offers an alternative to conventional respiratory therapies that focus on peripheral processes and intensive therapies. Central acting drugs offer a non-invasive treatment option with robust results. Overall, we find that setmelanotide improves hypercapnic chemosensitivity. These respiratory improvements are associated with weight loss. Our data would suggest that individuals unable to lose weight from setmelanotide would also fail to exhibit improvements in hypercapnic chemosensitivity.

## Supplementary Material

Supplementary Material

Appendix A. Supporting information

Supplementary data associated with this article can be found in the online version at doi:10.1016/j.resp.2024.104370.
