# Supplementary material for: The MC4R agonist, setmelanotide, is associated with an improvement in hypercapnic chemosensitivity and weight loss in male mice
Source: Respir Physiol Neurobiol. Author manuscript; Available in PMC 2025 Aug 5. (PMC12323632; doi:10.1016/j.resp.2024.104370)
Supplement: Supplementary Material [file NIHMS2100640-supplement-Supplementary_Material.pdf]

Supplemental Figures

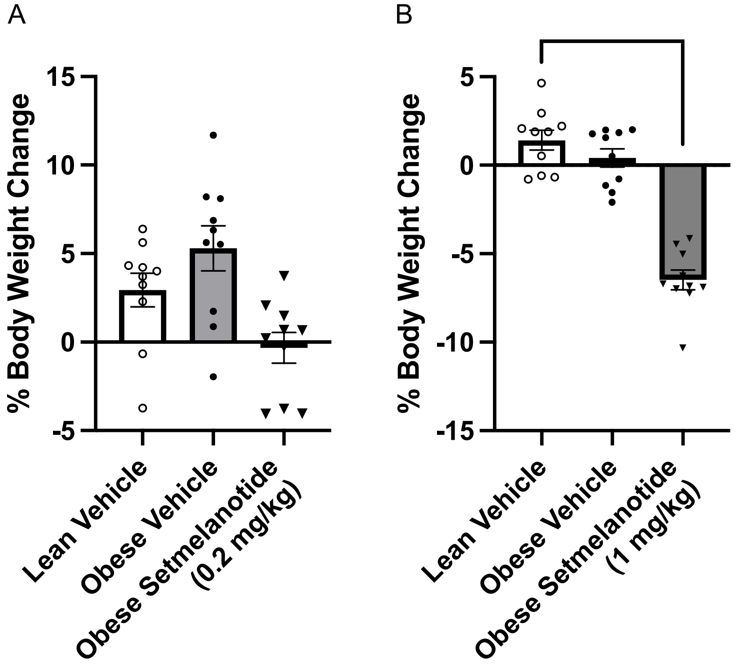

Supplemental Figure 1. Precent body weight change relative to body weights before treatment condition. A) Percent body weight change was not significant between groups. One-way ANOVA with Tukey's multiple comparison test. B) 1 mg/kg of Setmelanotide causes weight loss in mice. One-way ANOVA with Tukey's multiple comparison test for Lean Vehicle and Obese Setmelanotide (1 mg/kg), *** $p < 0.001$.

Setmelanotide does not affect glucose tolerance.

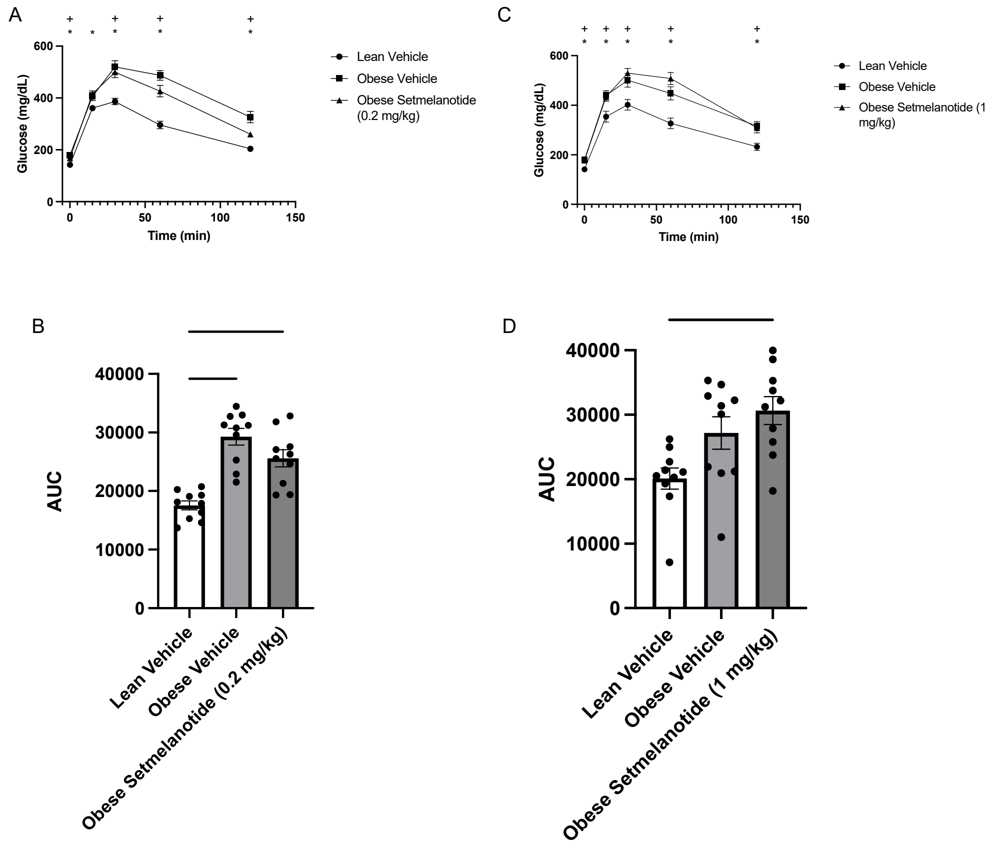

Supplemental Figure 2. Glucose Tolerance. **A**) 0.2 mg/kg setmelanotide does not affect glucose tolerance. Two-way ANOVA and a Tukey's multiple comparison test. Differences between the lean vehicle and the obese vehicle were seen (* $p < 0.05$) as well as the obese setmelanotide group and the lean vehicle (+ $p < 0.05$). However, there was no difference between obese vehicle and obese setmelanotide groups. **B**) Area under the curve (AUC) was calculated and analyzed for 0.2 mg/kg setmelanotide treatment using an ordinary one-way ANOVA **C**) Glucose tolerance does not change with 1.0 mg/kg setmelanotide treatment. Two-way ANOVA and a Tukey's multiple comparison. Differences between the lean vehicle and the obese vehicle were seen (* $p < 0.05$) as well as the obese setmelanotide group and the lean vehicle (+ $p < 0.05$). However, there was no difference between obese vehicle and obese setmelanotide groups. **D**) AUC was calculated and analyzed for 1.0 mg/kg setmelanotide treatment using an ordinary one-way ANOVA.

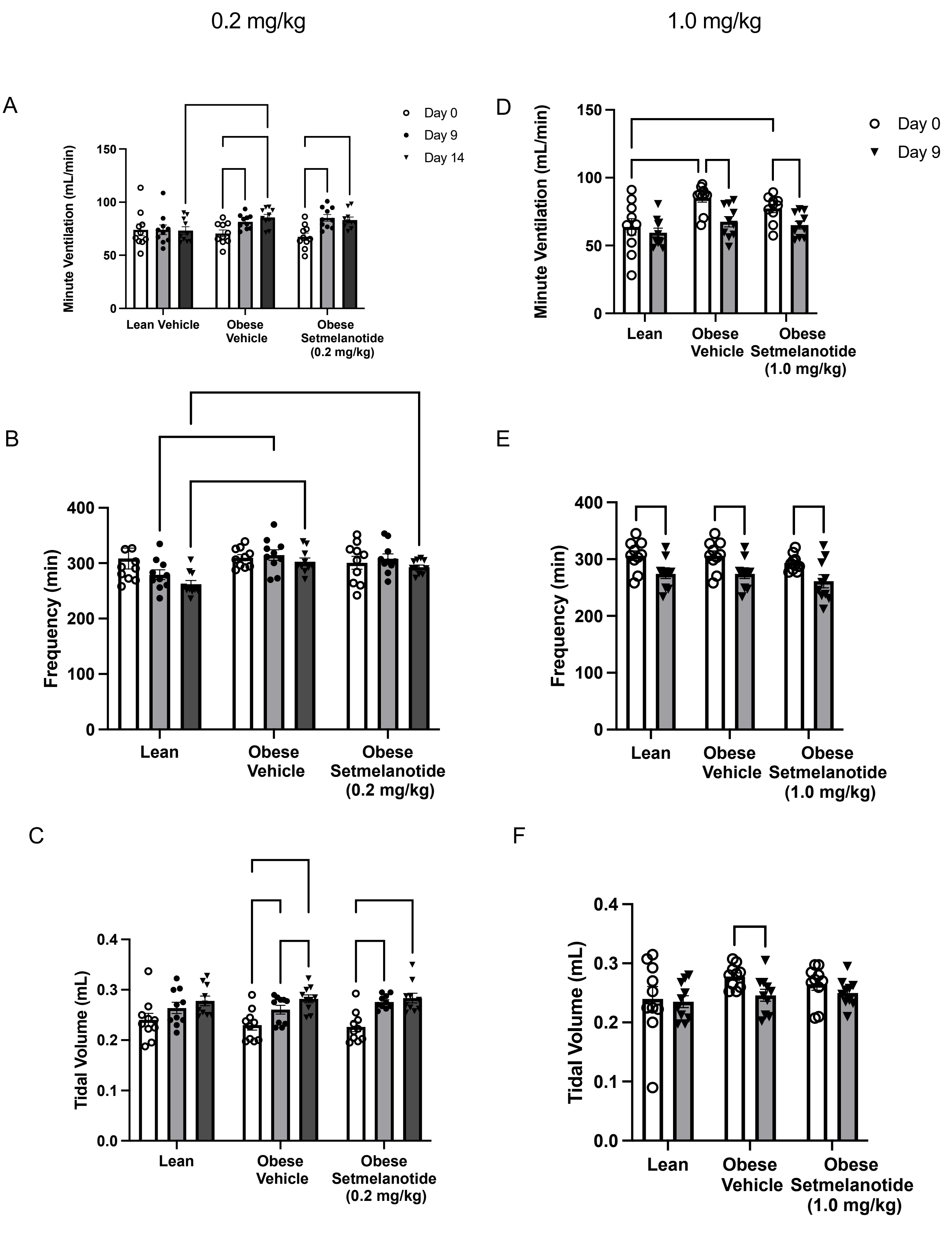
0.2 mg/kg
1.0 mg/kg
A
Day 0
Day 9
Day 14
Minute Ventilation (mL/min)
150
100
50
0
Lean Vehicle
Obese Vehicle
Obese Setmelanotide (0.2 mg/kg)
D
Day 0
Day 9
Minute Ventilation (mL/min)
Lean
Obese Vehicle
Obese Setmelanotide (1.0 mg/kg)
B
Frequency (min)
400
300
200
100
0
Lean
Obese Vehicle
Obese Setmelanotide (0.2 mg/kg)
E
Frequency (min)
Lean
Obese Vehicle
Obese Setmelanotide (1.0 mg/kg)
C
Tidal Volume (mL)
0.4
0.3
0.2
0.1
0.0
Lean
Obese Vehicle
Obese Setmelanotide (0.2 mg/kg)
F
Tidal Volume (mL)
Lean
Obese Vehicle
Obese Setmelanotide (1.0 mg/kg)

Supplemental Figure 3: **A.** Minute ventilation of 0.2 mg/kg setmelanotide treatment has a main effect time. Two-way ANOVA with Tukey's multiple comparison test, * $p < 0.05$, ** $p < 0.005$, and *** $p < 0.0005$. **B.** Frequency of 0.2 mg/kg setmelanotide treatment has a main effect of treatment as well as time. Two-way ANOVA with Tukey's multiple comparison test, * $p < 0.05$, ** $p < 0.01$, and *** $p < 0.001$. **C** Tidal volume 0.2 mg/kg setmelanotide treatment only has a main effect of time. Two-way ANOVA with Tukey's multiple comparison test, * $p < 0.05$, ** $p < 0.01$, and *** $p < 0.001$. **D**. Minute ventilation of 1.0 mg/kg setmelanotide treatment has a main effect of treatment as well as time. Two-way ANOVA with Tukey's multiple comparison test, * $p < 0.05$, ** $p < 0.005$, and *** $p < 0.0005$. **E**. Frequency of 1.0 mg/kg setmelanotide treatment has a main effect of treatment as well as time. Two-way ANOVA with Tukey's multiple comparison test, * $p < 0.05$, ** $p < 0.005$, and *** $p < 0.0005$. **F**. Tidal volume of 1.0 mg/kg setmelanotide treatment has a main effect of time. Two-way ANOVA with Tukey's multiple comparison test, * $p < 0.05$

Setmelanotide and Pair-feeding significantly reduce weight in mice.

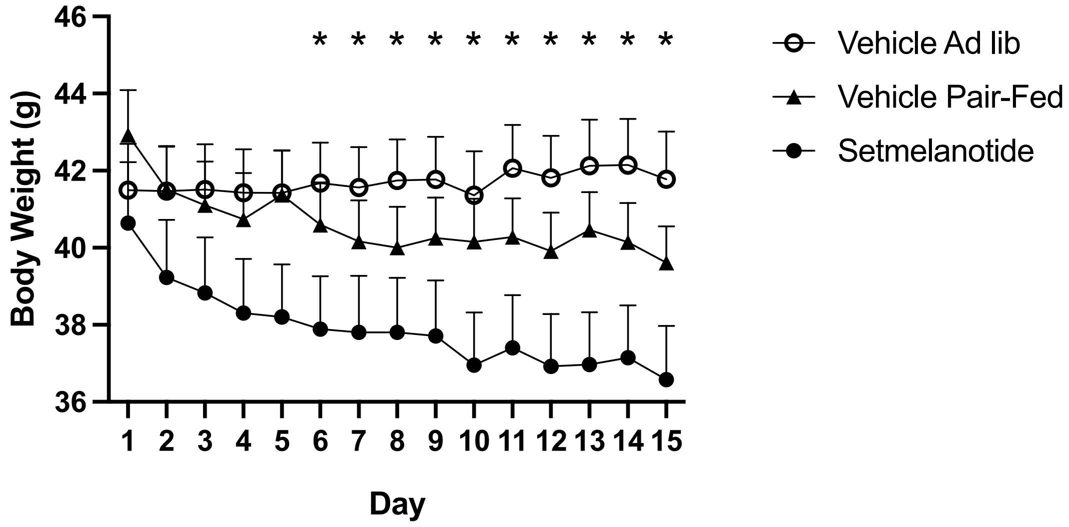

Supplemental Figure 4. Setmelanotide and pair-feeding causes weight loss in mice. Two-way ANOVA with Tukey's multiple comparison test for Vehicle *ad libitum* vs Setmelanotide, * $p < 0.05$

Setmelanotide does not affect eucapnic breathing.

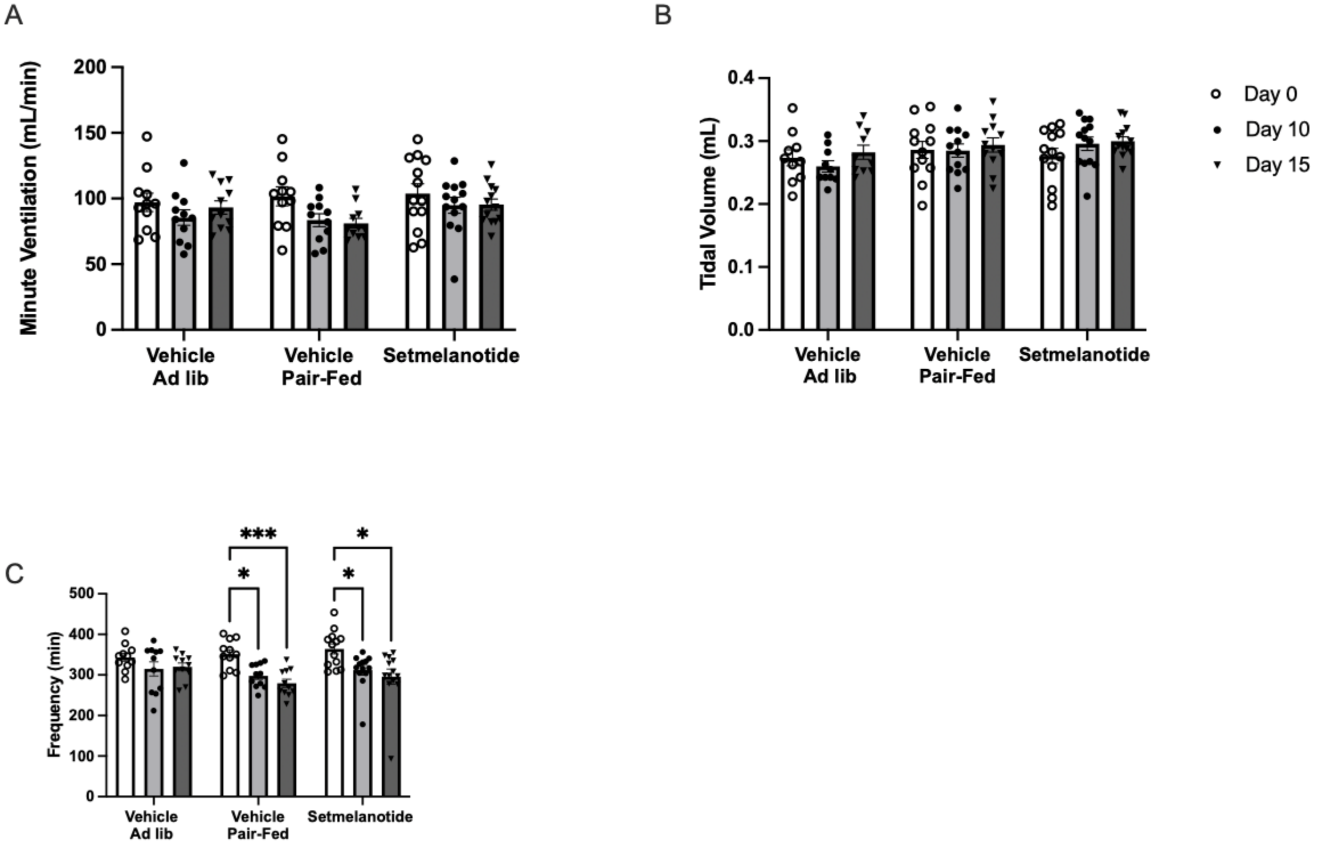

Supplemental Figure 5. **A**) Eucapnic breathing does not change with setmelanotide treatment (1 mg/kg). Eucapnic breathing from Day 0 to Day 15 was calculated for each group and is analyzed with a two-way ANOVA and a Tukey's multiple comparison test where no main effect and no interaction effect. **B**) Tidal volume does not change with setmelanotide treatment. Tidal volume from Day 0 to Day 15 was calculated for each group and is analyzed with a two-way ANOVA and a Tukey's multiple comparison test with no main effect seen. **C**) Frequency is not affected by setmelanotide treatment. Frequency from Day 0 to Day 15 was calculated for each group and is analyzed with a two-way ANOVA and a Tukey's multiple comparison test where only a main effect of time is seen.
